# Clinical Effectiveness of Bee Venom Acupuncture for Bone Fractures and Potential Mechanisms: A Narrative Overview

**DOI:** 10.3390/toxins16110465

**Published:** 2024-10-31

**Authors:** Jung-Hyun Kim, Byung-Kwan Seo

**Affiliations:** 1 Department of Acupuncture and Moxibustion Medicine, Kyung Hee University Hospital at Gangdong, Seoul 05278, Republic of Korea; dan_mi725@naver.com; 2 Department of Acupuncture and Moxibustion Medicine, College of Korean Medicine, Kyung Hee University Hospital at Gangdong, Kyung Hee University, Seoul 05278, Republic of Korea

**Keywords:** pain management, animal venoms, narrative review, treatment outcome, therapeutics

## Abstract

Bee venom acupuncture, a type of herbal acupuncture, combines the pharmacological actions of bioactive compounds from bee venom with the mechanical stimulation of meridian points. Bee venom acupuncture is gaining popularity, particularly in the Republic of Korea, primarily for pain relief of various conditions. This study aimed to summarize and evaluate the available evidence on the use of bee venom acupuncture for recovery after bone fractures. Electronic literature searches for experimental studies and clinical trials were conducted using the PubMed, China Academic Journals (CAJ), and OASIS databases. The search revealed 31 studies, of which six met our criteria. These studies demonstrated that bee venom acupuncture can be effective in treating bone fractures, suggesting a promising area for future research. However, evidence supporting its efficacy in this context is limited. Rigorous trials with large sample sizes and robust designs are needed to clarify the role of bee venom acupuncture for these indications. In addition, future studies should explore the optimal dosage and concentration of bee venom acupuncture.

## 1. Introduction

Bee venom has attracted considerable attention in complementary and alternative medicine (CAM) owing to its potential therapeutic applications in managing various medical conditions [1]. Used as a treatment since ancient times, bee venom is recognized for its pharmacological properties, which are attributed to a complex mixture of bioactive compounds, including peptides, enzymes, and amino acids. In recent years, interest in the application of bee venom in bone fracture healing has grown, leading to numerous clinical studies focused on evaluating its effectiveness [2].

The use of bee venom for bone fractures is based on its reported anti-inflammatory and analgesic effects, which may aid recovery and reduce the pain associated with musculoskeletal injuries. Traditional medical practices have employed bee venom in various forms, such as bee venom acupuncture (BVA), and contemporary research is now investigating its specific role in promoting bone regeneration and healing in clinical settings [3].

Fractures, classified by their characteristics and severity, can be categorized into types: simple (closed), compound (open), comminuted, and stress fractures. Each type poses unique challenges in regard to treatment and recovery [4]. The management of fractures has advanced significantly over time, with modern approaches primarily involving surgical intervention, immobilization, and rehabilitation. Surgical techniques, including internal fixation using plates and screws or external fixation devices, have become standard for complex fractures, aiming to restore anatomical alignment and promote healing [5].

Despite advancements in surgical techniques and postoperative care, conventional treatment methods have limitations. Surgical interventions carry risks of complications such as infections, non-union, and delayed healing. Furthermore, reliance on immobilization can cause stiffness and muscle atrophy, thereby prolonging recovery time. In addition, although pharmacological pain management is effective in the short term, it may lead to adverse effects and dependency, further complicating patient care [6].

These limitations have led patients and healthcare providers to explore CAM approaches as adjuncts to conventional treatments [7]. CAM therapies, such as bee venom therapy, acupuncture, and herbal medicine, are gaining popularity because of their potential to enhance healing, reduce pain, and improve overall patient outcomes [8]. The appeal of these therapies lies in their holistic nature, focusing on overall well-being while often minimizing the side effects commonly associated with conventional treatments.

As interest in CAM continues to grow, critically evaluating the efficacy and safety of these alternative approaches in bone fracture management has become essential. Gaining a deeper understanding of the reasons behind this shift toward CAM can provide valuable insights into patient preferences and the evolving landscape of therapeutic options in orthopedic care.

Although bee venom has been widely used in CAM, its specific role in early fracture healing, pain reduction, and functional recovery remains largely underexplored. Despite anecdotal reports and traditional practices suggesting its benefits, scientific evidence supporting the efficacy of bee venom in these contexts is limited and inconclusive [9].

The mechanisms through which bee venom may influence bone healing and pain management are not fully understood. Researchers hypothesize that bioactive compounds in bee venom, such as melittin and phospholipase A2, possess anti-inflammatory and analgesic properties that could promote tissue regeneration and modulate pain pathways [10]. However, robust clinical studies and rigorous scientific investigations are needed to establish a clear causal relationship between bee venom application and improved fracture healing outcomes [11].

Furthermore, the existing literature lacks standardized methodologies, comprehensive clinical trials, and long-term follow-up data, all of which are essential for validating the therapeutic claims associated with bee venom. Consequently, the integration of bee venom into conventional fracture management protocols remains limited, and healthcare practitioners may hesitate to recommend it without substantial evidence.

Although the potential of bee venom as a therapeutic agent in CAM has been acknowledged, the shortage of concrete evidence regarding its effectiveness in enhancing fracture healing and alleviating pain highlights the need for further research. Establishing a more definitive understanding of the role of bee venom in these areas may pave the way for its broader acceptance and application in clinical practice.

Therefore, the aim of this study was to evaluate data from experimental and clinical studies and to explore potential mechanisms for improving the understanding of the effectiveness of BVA in recovery after bone fractures.

## 2. Results Sections

### 2.1. Selected Studies

As of August 2024, a search of three databases—PubMed, CAJ, and OASIS—yielded 87 papers. After reviewing the titles, abstracts, and full texts of these papers, observational studies, animal studies, and those not focused on fractures or not employing bee venom therapy as an intervention were excluded. Ultimately, six papers were selected for analysis. All selected papers were from the OASIS database, and no papers meeting the inclusion criteria were identified in PubMed or CAJ. The details of the selected studies are presented in Table 1.

### 2.2. Favorable Effects of Bee Venom in Patients with Bone Fractures in Clinical Situations

The six studies mentioned above collectively demonstrated the potential of BVA therapies for treating various bone fractures. BVA has been found to be more effective than conventional acupuncture and other traditional Korean medicine treatments in managing thoracolumbar fractures, resulting in significant improvements in pain reduction and functional recovery. In addition, when combined with other traditional Korean medical treatments, BVA has revealed promising results in treating rib fractures, delayed union of distal phalanx fractures, and avulsion fractures of the proximal fifth metatarsal. These findings suggest that bee venom therapy may serve as a valuable adjunct to conventional treatments for bone fractures, offering potential benefits for pain management, healing, and overall patient outcomes.

### 2.3. Administration of Bee Venom as an Injectable

The administration of bee venom in injectable form is preferred in view of several critical advantages that enhance its therapeutic efficacy. Injectable bee venom ensures higher bioavailability of active components such as melittin and phospholipase A2, as oral administration typically leads to degradation of these compounds by gastric acids and digestive enzymes, significantly reducing their effectiveness [18]. Furthermore, the injectable route allows for immediate absorption into the bloodstream, providing a rapid therapeutic effect that is particularly beneficial for treating acute inflammatory conditions or pain [19]. In addition, injectables facilitate targeted delivery, enhancing efficacy in specific areas while minimizing systemic exposure and potential side effects. They also prevent gastrointestinal irritation and adverse reactions associated with oral administration, making injections safer for sensitive individuals [20]. Lastly, injectable formulations allow for precise dosing, ensuring consistent delivery of active compounds, which is challenging with oral formulations owing to variability in absorption influenced by factors such as food intake and individual metabolism [21].

## 3. Discussion

### 3.1. Potential Therapeutic Alternatives for Fracture Recovery

In addition to BVA, several other substances can be injected to promote bone regeneration after fractures. The potential advantages, limitations, and risks of these substances are as follows:

Bone morphogenetic proteins (BMPs), specifically BMP-2 and BMP-7, are growth factors that facilitate healing by promoting osteoblast differentiation and enhancing new bone formation [22,23]. However, BMPs have several limitations and risks that warrant careful consideration. Adverse effects associated with BMPs include complications such as ectopic bone formation, inflammatory responses, and malignancies [24]. In addition, the cost and availability of BMPs can pose significant barriers to their use, as they tend to be expensive and may not be accessible in all clinical settings. Furthermore, determining the optimal dose is crucial, as excessive administration can lead to adverse effects, whereas insufficient doses may fail to promote adequate healing [25].

Mesenchymal stem cells (MSCs) can be injected into fracture sites to promote bone regeneration by differentiating into osteoblasts and secreting various growth factors that aid in healing [26]. However, several significant risks and limitations of MSCs must be addressed before their clinical application. One major concern is the potential for tumorigenicity, particularly if cells are inadequately characterized or improperly administered [27]. In addition, the efficacy of MSCs can vary widely depending on their source, such as whether they are derived from bone marrow or adipose tissue, as well as the specific health condition of the patient [28]. Moreover, the use of allogeneic MSCs may trigger an immune response, potentially leading to rejection or other complications [29].

Hydroxyapatite is a biocompatible material that can be injected to promote healing. It provides a bioactive scaffold that supports cell attachment and growth, thereby enhancing osteoconductivity [30]. Although generally considered biocompatible, hydroxyapatite has several limitations that may impact its clinical use [31]. Reports of local inflammation and adverse tissue reactions have been noted in association with its use [32]. In addition, the lower mechanical strength of hydroxyapatite compared with that of natural bone may limit its effectiveness in load-bearing applications [33]. Furthermore, because hydroxyapatite is non-resorbable, it can cause long-term foreign body reactions in some patients [34].

Sodium hyaluronate promotes healing by improving the viscosity and elasticity of the extracellular matrix, aiding in tissue regeneration, and facilitating the delivery of growth factors [35,36]. However, its clinical application presents certain challenges primarily owing to its short duration of action, as its benefits tend to diminish over time, necessitating repeated injections for sustained relief [37,38]. Furthermore, the response to sodium hyaluronate can vary significantly among patients and is influenced by individual biological factors, which may complicate treatment outcomes [39].

In addition to the substances mentioned above, bee venom demonstrates significant analgesic effects, effectively managing pain by modulating pain pathways and offering a natural alternative to traditional analgesics. Moreover, bee venom generally has fewer side effects compared with synthetic drugs, although allergic reactions may occur in some individuals. Its multitarget mechanisms, attributed to various bioactive compounds, enhance its versatility in treating diverse conditions. Bee venom can also serve as an adjunct therapy, improving overall treatment efficacy while potentially reducing the required dosages of conventional medications.

### 3.2. Potential Mechanisms Through Which Bee Venom May Aid in Fracture Recovery

#### 3.2.1. Melittin

Melittin enhances the proliferation of osteoblasts, which are crucial for bone formation. This stimulation can lead to an increased number of osteoblasts at the fracture site, thereby promoting the healing process of the bone. Mellitin may activate various signaling pathways crucial for osteoblast function. For instance, it can influence the Wnt/β-catenin signaling pathway, which plays a significant role in osteoblast differentiation and proliferation [40].

Melittin may also enhance the synthesis of extracellular matrix components, such as collagen. A robust extracellular matrix is vital for osteoblast attachment and function, facilitating improved bone repair. By reducing the levels of pro-inflammatory cytokines, melittin creates a more favorable environment for osteoblast activity. As inflammation can hinder bone healing, its reduction allows osteoblasts to function more effectively [41].

Melittin possesses anti-apoptotic properties, helping to prevent programmed cell death in osteoblasts. By maintaining a healthy population of these cells, melittin supports ongoing bone formation and repair. In addition, melittin may promote angiogenesis, the formation of new blood vessels, at fracture sites. An improved blood supply ensures that osteoblasts receive adequate nutrients and oxygen, which are critical for their proliferation and activity [42].

#### 3.2.2. IL-6 and IL-1α

Interleukin (IL)-6 signaling is crucial for bone repair, promoting anti-inflammatory and pro-regenerative processes. Inhibition of IL-6 during the early inflammatory phase can result in delayed healing owing to diminished immune cell recruitment and impaired bone regeneration [43]. Specifically, IL-6 stimulates the expression of regeneration genes in the periosteum, enhances cell proliferation, and reduces apoptosis, all of which are vital for effective fracture healing [44].

IL-1α is known to initiate inflammatory responses that can influence bone remodeling. Although specific studies on the role of IL-1α in fracture healing are limited, its role in inflammation suggests a potential positive contribution to the healing process. IL-1α is involved in the inflammatory response following bone injury, promoting the secretion of IL-1β and IL-18, which are critical for fracture healing. The absence of IL-1 signaling, as demonstrated in knockout models, leads to delayed fracture healing, highlighting its importance in the inflammatory phase of recovery [45]. In addition, IL-1α interacts with histone acetyltransferases, indicating a nuclear role that may affect gene expression related to inflammation and healing [46].

Both IL-6 and IL-1α can positively influence bone fracture recovery by modulating inflammatory responses and promoting regenerative pathways, underscoring the therapeutic potential of bee venom in enhancing the activities of these cytokines.

#### 3.2.3. IL-1 and TNF-α

Bee venom stimulates osteoblast proliferation and differentiation, counteracting the effects of IL-1 and tumor necrosis factor (TNF)-α, which inhibit osteoblast activity and promote apoptosis in these cells [47]. This enhanced osteoblast activity contributes to increased bone formation and regeneration.

The ability of bee venom to modulate the balance between pro-inflammatory and anti-inflammatory cytokines is crucial for effective bone healing [48]. By downregulating IL-1 and TNF-α, bee venom may create an environment that supports bone regeneration while minimizing the detrimental effects of inflammation.

#### 3.2.4. TGF-β and VEGF

Transforming growth factor (TGF)-β is a critical cytokine in bone healing, as it promotes osteoblast differentiation and bone matrix formation. Studies indicate that components of bee venom can stimulate the production of TGF-β, thereby enhancing the bone healing process by promoting the proliferation and differentiation of osteoblasts. In addition, this cytokine plays a role in recruiting MSCs to fracture sites, facilitating regeneration [49].

Vascular endothelial growth factor (VEGF) is essential for angiogenesis, a process crucial for supplying nutrients and oxygen to healing bone tissue. Bee venom has been shown to enhance VEGF expression, thereby supporting the formation of new blood vessels in the bone environment. Increased angiogenesis improves the delivery of essential nutrients and growth factors, further aiding in bone regeneration [50].

The interplay between TGF-β and VEGF is crucial for effective bone healing. TGF-β not only promotes osteoblast activity but also enhances VEGF expression, creating a synergistic effect that supports both bone formation and vascularization [51]. The ability of bee venom to enhance both pathways may lead to improved outcomes in bone regeneration.

By modulating TGF-β levels, bee venom may help inhibit osteoclast differentiation, which is often stimulated by pro-inflammatory cytokines [52]. This inhibition can reduce bone resorption, creating a more favorable balance between bone formation and resorption during the healing process [53].

## 4. Conclusions

BVA has shown promising outcomes in treating various types of bone fractures, including thoracolumbar compression, rib, and delayed union of distal phalanx fractures. The observed improvements in pain reduction, functional recovery, and overall healing outcomes highlight the potential benefits of BVA as an adjunct to conventional treatments.

The mechanisms underlying the therapeutic effects of BVA are complex and involve multiple biological pathways. BVA may influence bone healing by modulating inflammatory responses, promoting osteoblast activity, enhancing angiogenesis, and regulating cytokine levels. Together, these mechanisms create a favorable environment for bone regeneration and repair.

Despite these encouraging findings, further research is needed to establish the definitive role of BVA in treating bone fractures. Rigorous clinical trials with larger sample sizes, standardized methodologies, and long-term follow-ups are essential to validate the efficacy and safety of BVA in diverse patient populations. In addition, investigating the optimal dosage, treatment regimen, and potential combination therapies with conventional treatments could further refine the application of BVA in clinical practice.

## 5. Methods

### 5.1. Data Sources

Electronic literature searches were conducted using the Pub-Med (https://pubmed.ncbi.nlm.nih.gov/, accessed on 17 August 2024), China Academic Journal (CAJ, https://www.cnki.net/index/, accessed on 17 August 2024), and OASIS databases (https://oasis.kiom.re.kr/index.jsp, accessed on 17 August 2024) to identify experimental research and clinical trials on BVA for bone fractures. In addition, relevant papers were sought in two prestigious Korean journals: *The Journal of the Korean Society for Acupuncture and Moxibustion* and *The Journal of Korean Oriental Medicine*. We used the keywords (“Bone fractures”; “Fractures, Compression”; and “Bee venom”) and free words (“Fractures”; “Bone Loss”; “Bone Losses”; “Compression Fracture”; “Fracture, Compression”; “Compression Fractures”; “Pharmacopuncture”; and “Acupoint injection”) to screen these databases. The reference lists of all identified articles underwent additional manual searches.

### 5.2. Categorization of Studies

**Figure 1:**
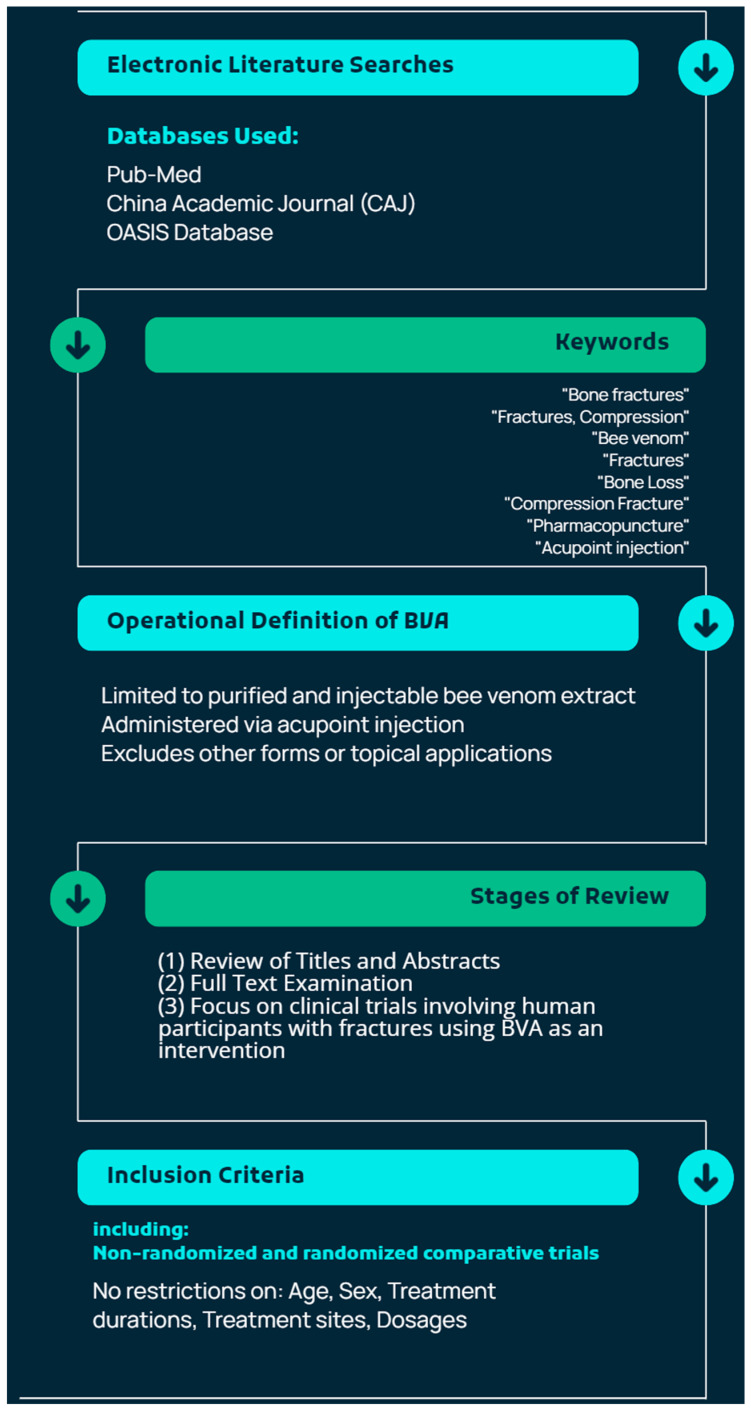
Summary of retrieval procedures for articles involving bee venom acupuncture (BVA) for bone fracture treatment.

In this review, BVA is limited to the form of bee venom that has been purified and prepared as an injectable solution and administered through injection into acupoint locations. Cases involving the injection of bee venom in other forms or its application topically were excluded from this review. In the first stage, the titles and abstracts of the identified articles were reviewed. In the second stage, the full texts were examined to select studies that were clinical trials conducted on human participants, specifically targeting patients with fractures and using BVA as an intervention. Among the retrieved articles, we aimed to analyze all clinical trials, including both non-randomized and randomized comparative clinical trials that employed BVA as an intervention for patients with fracture injuries, without imposing restrictions on age, sex, duration, or injury site in the study population. In addition, regarding the use of BVA as an intervention, we did not impose restrictions on dosages, treatment sites, or methodologies, thereby encompassing all therapeutic approaches that involved components of bee venom. Whole retrieval procedures are demonstrated in Figure 1.

### 5.3. AI Language Model Usage

While preparing the manuscript, various artificial intelligence (AI) tools were essential for ensuring the accuracy and clarity of the English language used. Grammarly (https://www.grammarly.com/, accessed on 19 September 2024), an advanced tool powered by AI, was brought into play for thorough inspections of grammar, spelling, and punctuation. The Hemingway Editor (http://www.hemingwayapp.com/, accessed on 19 September 2024) was applied to increase the understandability of the text and to lessen the intricacy of sentences. LanguageTool (https://languagetool.org/, accessed on 20 September 2024), adept at performing grammar and spelling checks that are context-specific across a diversity of English dialects, was implemented as well. Ginger (https://www.gingersoftware.com/, accessed on 21 September 2024), another tool with AI, was employed for the restructuring of sentences and the enhancement of language flow. Slick Write (https://www.slickwrite.com/, accessed on 21 September 2024) was brought in to carry out an analytical examination of sentence construction and the overarching writing style. These tools were used in a repetitive manner throughout the writing process to uphold a high caliber of academic English.

## 6. Future Directions

The fact that only the OASIS database yielded relevant results, while other major databases did not, suggests a potential narrow scope of research or a specific regional emphasis within the field. The reliance on the OASIS database can be justified by its specialization in curating materials that focus on particular topics or regions, which may align closely with the research objectives. Additionally, OASIS is known for its rigorous vetting process, ensuring that the included materials are of high quality and reliability, essential for academic rigor. Furthermore, it may contain literature on niche subjects that are underrepresented in other databases, providing critical insights relevant to the study. Lastly, if the research has a geographical or community focus, OASIS is likely to offer a more comprehensive collection of data that reflects local perspectives and issues, thereby enhancing the contextual relevance of the findings.

The lack of findings from more internationally recognized databases highlights a limitation in this review that could raise concerns about selection bias. By relying primarily on the OASIS database, there is a risk that the research may not fully capture the breadth of available literature, particularly studies that might provide contrasting or complementary perspectives from a wider international context. Acknowledging this limitation is crucial, as it underscores the need for caution in generalizing the findings. Future research could benefit from a more comprehensive approach that includes a broader range of databases, thereby enhancing the credibility of the review and ensuring a more balanced representation of the field. This would help mitigate potential biases and provide a more nuanced understanding of the topic.

## Figures and Tables

**Table 1:** Summary of included studies.

First Author (Year)	Study Design	Groups	Primary Outcome	Main Results	Adverse Effects	Formulations * /Effective Dosage	Authors′ Conclusions
Lee et al. (2002) [12]	Observational study	Experimental: BVA + TKM treatments for 16 patients, Control: AT for 23 patients	Young’s Grade	(1) 87.5% of patients in the BVA group rated their outcomes as “Good” or higher, compared with 47.8% in the AT group. (2) The BVA group demonstrated greater improvement across all clinical symptom grades. (3) 50% of the BVA group achieved over 80 degrees of improvement in lumbar flexion, compared with 21.7% in the AT group.	No detrimental changes in serum aspartate aminotransferase, alanine aminotransferase, gamma-glutamyl transpeptidase, blood urea nitrogen, or creatinine levels were observed in either group.	3000:1 dilution/1.0 mL injection	BVA may be more effective than AT for treating thoracolumbar compression fractures in clinical practice.
Yang et al. (2008) [13]	Observational study	Experimental: BVA + TKM treatments for 15 patients, Control: AT for 13 patients	(1) VAS (2) ODI	(1) The study had a higher proportion of females, with an average age in the 70s. (2) Accidental falls were the most common cause of injury. (3) Both groups showed significant improvement in VAS and ODI scores from baseline to final evaluation. (4) The BVA group demonstrated significantly better VAS and ODI scores compared with the AT group after treatment.	Not reported	3000:1 dilution/0.2 mL injection	BVA is effective for relieving symptoms associated with thoracolumbar compression fractures.
Oh et al. (2015) [14]	Case study	Single patient (63-year-old female)	(1) AHS (2) VNRS (3) ROM of the ankle joint	After treatment, the American Orthopedic Foot and Ankle Society AHS score improved from 18 to 71, the VNRS score decreased from 8 to 3, and the ROM of the ankle joint improved.	Not reported	20,000:1 dilution/0.2 mL injection	BVA and Danggwisu powder (medication) are effective treatments for lateral malleolus avulsion fractures.
Ahn et al. (2019) [15]	Case study	Single patient (46-year-old)	NRS	The patient was initially undiagnosed with a rib fracture on chest radiography but was subsequently diagnosed using ultrasound. After four weeks of integrative Korean medical treatments and 18 sessions of ultrasound-guided bee venom pharmacopuncture, pain on the NRS decreased from 8 to 2. Pain relief was immediate following BVA and lasted for three hours.	Not reported	10,000:1 dilution/0.1 mL injection	Ultrasound-guided essential BVA may act as a beneficial treatment method for rib fractures.
Park et al. (2019) [16]	Case study	Single patient (4th toe distal phalanx fracture)	(1) NRS (2) Morphological changes on radiography	33 sessions of combined treatments, including acupuncture, cupping, BVA, moxibustion, and herbal medicine. Improvement in the delayed union of the fracture was observed, along with a reduction in pain as measured using the NRS.	Not reported	10,000:1 dilution/0.1 mL injection	Traditional Korean medicine treatment may be effective for delayed union of fractures; however, further clinical studies are needed to confirm these findings.
Ahn et al. (2021) [17]	Case study	Single patient (proximal fifth metatarsal avulsion fracture)	(1) NRS (2) Morphological changes on radiography	The patient received a combination of acupuncture, BVA, moxibustion, cupping, and herbal medicine. Following treatment, improvement in the avulsion fracture was observed, along with reduced pain as measured using the NRS.	Not reported	10,000:1 dilution/0.1 mL injection	Traditional Korean medical therapy may be an effective treatment option for avulsion fractures of the proximal fifth metatarsal.

* Dried bee venom extracts are diluted within normal saline solution. AHS: Ankle-hindfoot scale; NRS: Numerical rating scale; VNRS: Verbal numerical rating scale; ROM: Range of motion; TKM: Traditional Korean medicine; VAS: Visual analogue scale; ODI: Oswestry disability index; AT: Acupuncture treatment; BVA: Bee venom acupuncture.

## Data Availability

No new data were created or analyzed in this study. Data sharing is not applicable to this article.
